# Withdrawal of Gamma-Hydroxybutyrate in a Saudi Male Patient: A Case Report

**DOI:** 10.7759/cureus.32298

**Published:** 2022-12-07

**Authors:** Ibtihal M Alattas, Sultan A Alwajeeh, Osama O Alamoudi, Abdulmajeed H Alzahrani, Badria A Alhatali

**Affiliations:** 1 Emergency Medicine, King Abdulaziz University Faculty of Medicine, Jeddah, SAU; 2 Psychiatry, Al Amal Hospital, Ministry of Health, Jeddah, SAU; 3 Nursing, King Khalid University Hospital, Jeddah, SAU; 4 Emergency Medicine/Toxicology, Poison Control Section, Ministry of Health, Muscat, OMN

**Keywords:** date rape drug, ghb intoxication, cns depressant, ghb withdrawal, ghb, gamma-hydroxybutyrate

## Abstract

Gamma-hydroxybutyrate (GHB) is a central nervous system (CNS) depressant with limited clinical use but has been misused in the last few decades. During intoxication, the patient may develop CNS depression and may have agitation, while during withdrawal, the patient can present with severe agitation or delirium. Here, we report the case of a 30-year-old Saudi male patient who was brought by his brother to the emergency department (ED) with agitation and delirium. The patient’s friend stated the patient had been misusing GHB mixed with alcohol for the last seven months, with the last use occurring 24 hours before the ED presentation. The patient was put on a five-point restraint for safety concerns and received supportive therapy. After two days of admission, the patient completely recovered. As the patient provided a limited history of his GHB misuse, the clinician lacked sufficient information to determine whether the patient was intoxicated or withdrawing. Clinicians in Saudi Arabia need to be highly suspicious of GHB misuse when treating patients with drug intoxication or withdrawal.

## Introduction

Gamma-hydroxybutyrate (GHB) is a central nervous system (CNS) depressant and an analog for gamma-aminobutyric acid (GABA) [1], which is found naturally in small amounts in the human body [2]. GHB was discovered in 1960 and used along with its analogs, gamma-butyrolactone (GBL) and 1,4-butanediol (BD), as an anesthetic adjunct, treatment for sleep disturbance, dietary supplement for bodybuilders, and an antidepressant and adjunct treatment for alcohol detoxification and withdrawal [3]. Due to its euphoric effects, it was rapidly used as a “club drug” and gained popularity for misuse in the last three decades. It has been well-documented in several countries, such as Australia, the United States, and several European countries [4]. GHB is used in drug-facilitated sexual assaults as it can induce coma and profound amnesia. GHB has a narrow therapeutic index, with a maximum serum concentration occurring after 20-40 minutes, and has a half-life of 30-50 minutes which makes it easy to cause significant intoxication [2,5]. Even though the drug is a CNS suppressant that leads to a decreased level of consciousness, it can cause agitation that mimics the effects of amphetamine and methamphetamine intoxication. It also mimics other syndromes associated with antipsychotic and antidepressant medications, such as serotonin and neuroleptic malignant syndromes [6]. Unfortunately, the diagnosis of intoxication can be challenging because the drug is not detectable in the standard urine drug screening. In addition, the drug has been used as a club drug at parties [7] and recreationally as it has anxiolytic, euphoric, and some positive effects on libido [8].

Unlike other drugs used to facilitate sexual assaults, e.g., ketamine and Rohypnol, GHB has been linked to life-threatening severe withdrawal symptoms such as tremors, agitation, delirium, anxiety, hypertension, and coma that usually force users to seek medical treatment [2,4,9-11]. GHB withdrawal delirium is reported to be more common (12%) than alcohol withdrawal delirium (5%) and similarly associated with autonomic hyperarousal, although it is more severe and accompanied by psychosis [11]. Both intoxication and withdrawal syndromes have been linked to death [12,13]. The GHB withdrawal symptoms may start as early as 24 hours after cessation of the medication and may last up to a week. There is no standardized protocol to treat GHB withdrawal; however, most case reports used supportive care with high doses of benzodiazepines [2,14-17].

GHB misuse has never been reported in Saudi Arabia. We present a case of GHB withdrawal that started after one day after the last dose of GHB. This case highlights the possibility of encountering GHB intoxication or withdrawal in emergency departments (EDs) in Saudi Arabia.

## Case presentation

A healthy 30-year-old male, not on any medication, arrived at the ED at an addiction detoxification facility in Saudi Arabia with complaints of insomnia. On the first visit, he admitted using alcohol and what he called “G fluid” (gamma-hydroxybutyric acid) to self-medicate his symptoms. During the second visit, history was provided by the patient’s friend, who was abusing the same substance, along with the patient, for recreational purposes.He reported the patient had been adding a few drops of the GHB solution (unknown concentration) to a glass of ethanol two to three times per week for the last seven months. At the first visit, he was evaluated in the ED and a prescription of 30 mg mirtazapine was issued for his insomnia with a follow-up appointment in the psychiatry clinic. His urine drug screen was negative for cannabis, benzodiazepines, amphetamines, methamphetamines, and opiates. During the second visit, he was noticed to be agitated, aggressive, and speaking incoherently. Upon examination, the patient was found to be disoriented, partially responsive (Glasgow Coma Scale score of 7), and his vital signs were within normal range. He was speaking incoherently, wandering around with closed eyes to avoid black magic, as he stated. The rest of the mental examination was difficult to perform. He was kept on a five-point restraint for his safety. His vital signs were within normal limits. The patient received intravenous fluid (normal saline). His urine drug screen on the second visit and laboratory investigations were unremarkable.

During the six-hour observation period, he started to experience short intermittent episodes of extreme agitation and shouting lasting for one minute every 10-15 minutes, followed by complete calmness. However, his speech remained incoherent. He required one dose of intramuscular 5 mg haloperidol to control his agitation. Eight hours after arrival at the ED, he gradually became responsive and slept for 16 hours. Twenty-four hours after the ED visit, he regained full consciousness and could not remember the course of his illness in the hospital. He admitted mixing ethanol and GHB for recreational purposes and self-treating his recurrent insomnia. On day three of admission, he was alert and oriented to time, place, and person. He was discharged home with instructions to avoid using GHB and was educated about the danger of such a practice.

## Discussion

GHB has been increasingly misused for recreational purposes, a practice that, to our knowledge, has never been reported in the Gulf Cooperation Council countries. Our patient admitted using GHB for recreational purposes for seven months which might address a new trend in drug misuse in the region. He suffered from withdrawal symptoms that manifested 24 hours after his last dose was ingested and recovered completely within three days. The withdrawal symptoms of GHB develop after cessation of its use and last typically from two days to 31 days [18]. Tolerance and dependence are well described in frequent GHB users who are susceptible to withdrawal after discontinuation or reduction of the dose [3]. It is relatively mild and associated with autonomic instability and prolonged psychotic syndrome [17]. Our patient behaved in a similar pattern without developing autonomic instability. To establish the diagnosis of withdrawal, physicians need to have a high index of suspicion. A review of the clinical manifestation of GHB withdrawal highlighted the possibility of mislabeling the symptoms as alcohol withdrawal due to similarity in the presentation, although with GHB withdrawal symptoms occurred earlier with delirium being associated with severe dependence [19]. One of the challenges in the diagnosis is that the routine urine drug screen does not detect GHB, and a special screening test must be requested. It is crucial to know the time of the last dose used which might help in differentiating between GHB intoxication and withdrawal. This is important as administering benzodiazepines in an intoxicated patient with two CNS suppressants might not be a good strategy in treating such patients; thus, a period of observation is warranted. In general, the treatment of GHB withdrawal is supportive with close monitoring of the vital signs and associated symptoms. Hospital admission is required, especially when patients experience delirium, agitation, or autonomic instability. Previous case reports suggested treating agitation or delirium with a high dose of benzodiazepines [2,18,19]. In refractory cases, barbiturates and propofol have been used [17].

We present the first GHB withdrawal case to be reported from Saudi Arabia. We did a MEDLINE search using PubMed for the MeSH term “Saudi Arabia” AND gamma hydroxybutyrate, and we did not retrieve any published articles about the topic in this area.

## Conclusions

GHB is one of the recreational drugs that is popular in some countries. Although there is a lower likelihood of encountering a patient misusing GHB compared with other commonly misused substances in Saudi Arabia, it is, nonetheless, a possibility. We report a case of GHB withdrawal in Saudi Arabia to increase awareness among psychiatrists, toxicologists, and emergency physicians about GHB misuse, withdrawal symptomatology, and management. Because GHB is not detected in routine urine testing, physicians must have a strong suspicion of both intoxication and withdrawal while treating patients with suspected drug misuse.
